# Artificial intelligence for waste management in smart cities: a review

**DOI:** 10.1007/s10311-023-01604-3

**Published:** 2023-05-09

**Authors:** Bingbing Fang, Jiacheng Yu, Zhonghao Chen, Ahmed I. Osman, Mohamed Farghali, Ikko Ihara, Essam H. Hamza, David W. Rooney, Pow-Seng Yap

**Affiliations:** 1 Department of Civil Engineering, Xi’an Jiaotong-Liverpool University, Suzhou, 215123 China; 2 School of Chemistry and Chemical Engineering, Queen’s University Belfast, David Keir Building, Stranmillis Road, Belfast, BT9 5AG Northern Ireland UK; 3 Department of Agricultural Engineering and Socio-Economics, Kobe University, Kobe, 657-8501 Japan; 4 Department of Animal and Poultry Hygiene & Environmental Sanitation, Faculty of Veterinary Medicine, Assiut University, Assiut, 71526 Egypt; 5 Electric and Computer Engineering Department, Aircraft Armament (A/CA), Military Technical College, Cairo, Egypt

**Keywords:** Artificial intelligence, Waste management, Chemical analysis, Optimization, Cost efficiency

## Abstract

The rising amount of waste generated worldwide is inducing issues of pollution, waste management, and recycling, calling for new strategies to improve the waste ecosystem, such as the use of artificial intelligence. Here, we review the application of artificial intelligence in waste-to-energy, smart bins, waste-sorting robots, waste generation models, waste monitoring and tracking, plastic pyrolysis, distinguishing fossil and modern materials, logistics, disposal, illegal dumping, resource recovery, smart cities, process efficiency, cost savings, and improving public health. Using artificial intelligence in waste logistics can reduce transportation distance by up to 36.8%, cost savings by up to 13.35%, and time savings by up to 28.22%. Artificial intelligence allows for identifying and sorting waste with an accuracy ranging from 72.8 to 99.95%. Artificial intelligence combined with chemical analysis improves waste pyrolysis, carbon emission estimation, and energy conversion. We also explain how efficiency can be increased and costs can be reduced by artificial intelligence in waste management systems for smart cities.

## Introduction

Rapid urbanization, population growth, and economic development have increased waste generated worldwide in recent years. According to the latest statistics, 2.01 billion tonnes of municipal solid waste was generated globally in 2016. This figure is expected to increase to 3.4 billion tonnes by 2050 (Kaza et al. 2018). Unfortunately, 33% of solid waste is managed correctly and disposed of in illegal dumpsites or unmonitored landfills (Kaza et al. 2018). Improper waste disposal poses many environmental and health risks, such as groundwater contamination, land degradation, increased cancer incidence, child mortality, and congenital disabilities (Triassi et al. 2015). In the past, waste management practices were more rudimentary, with a small group of individuals collecting garbage from the streets and depositing it in designated areas (Brancoli et al. 2020). Once the trucks were full, the waste was left in these designated areas. However, with the advent of artificial intelligence, the waste management industry is experiencing significant transformation toward achieving sustainability and profitability.

Artificial intelligence is a rapidly advancing technology that is gaining popularity in various industries, particularly waste management (Abdallah et al. 2020). The incorporation of artificial intelligence and robotics in the design and operation of urban waste treatment plants can revolutionize how solid waste is managed, leading to increased operational efficiency and more sustainable waste management practices (Goutam Mukherjee et al. 2021; Yigitcanlar and Cugurullo 2020). Several developed countries, including Austria, Germany, New Zealand, the USA, the UK, Japan, Singapore, Switzerland, South Korea, and Canada, have already begun to adopt artificial intelligence technologies to maximize resource utilization, efficiency, and recycling opportunities throughout the solid waste management cycle (Soni et al. 2019). Artificial intelligence technologies, particularly for sorting and treating solid waste, are increasingly critical in waste management (Andeobu et al. 2022; Wilts et al. 2021).

Therefore, artificial intelligence is critical in developing sustainable waste management models, particularly for transitioning to a “zero waste circular economy” while considering social, economic, and environmental factors (Osman et al. 2022). Waste management should be considered when examining the problems facing different geographic areas and economic sectors, including smart cities. For instance, researchers have proposed various models for sustainable waste management, such as a model for megacities that considers waste treatment, recycling, and reuse options (Liamputtong 2009). To choose the best location for solid waste management system components, another model was developed by researchers that take into account uncertain waste generation rates, facility running costs, transportation costs, and revenue (Chadegani et al. 2013). Based on the England panel data, Liu et al. (2017) investigated data on landfill, waste management, and environmental safety in England, including the reasons for illegal dumping (Goutam Mukherjee et al. 2021; Yigitcanlar and Cugurullo 2020). In addition, Zhang et al. (2019) emphasized the need for a new school of management thought to transition to a “zero waste circular economy.”

**Fig. 1:**
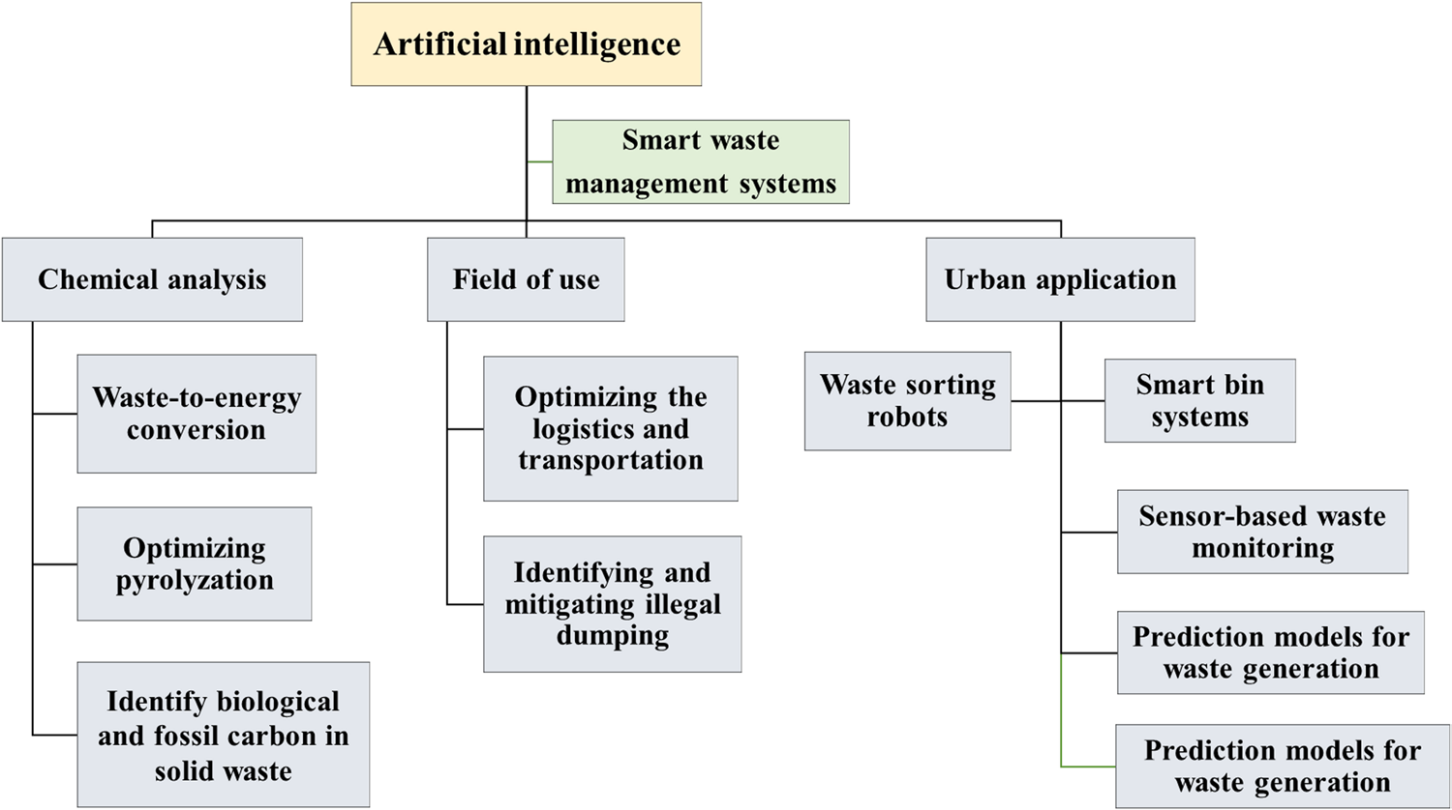
Application of artificial intelligence in waste management. The figure illustrates five key aspects: waste type and generation, the use of artificial intelligence in waste management, artificial intelligence-based optimization of waste transportation, the role of artificial intelligence in detecting and reducing illegal dumping and waste treatment practices, and the use of artificial intelligence to analyze the chemical composition of waste. This optimized representation provides a clear and concise overview of the main themes discussed in this review, highlighting the potential of artificial intelligence to revolutionize waste management practices

To summarize, this paper provides an overview of waste types, their generation, and associated issues, as well as explores various applications of artificial intelligence in waste management. These applications include intelligent bin systems, waste-sorting robots, sensor-based waste monitoring, and predictive models of waste generation. Additionally, the paper discusses how artificial intelligence can help monitor and track waste materials throughout the recycling process, optimize the logistics and transportation of recycled waste, identify and reduce illegal dumping and waste treatment practices, and analyze the chemical composition of waste. One of the unique contributions of this paper is the combination of artificial intelligence and waste chemical analysis to improve the process of converting waste into energy. Figure 1 shows the key concepts of artificial intelligence in waste management and the content of this article.

## Waste types and production

Waste is a major environmental issue due to its potential to contaminate air, water, and soil. Its generation is mainly attributed to human activities such as industrial production, construction, agricultural activities, pollution emissions, consumption, and waste disposal (Chen et al. 2023; Ukaogo et al. 2020). This can lead to environmental pollution, health risks, economic losses, and the loss of resources and costs associated with waste management. To address these issues, governments and organizations have implemented waste management strategies such as recycling, composting, reusing, using renewable energy, and adopting green technologies. Moreover, public education and awareness campaigns can help reduce the amount of waste generated and encourage individuals to make more sustainable choices (Osman et al. 2023).

Waste classification principles vary and may involve categorization based on the waste’s material, state, or source (Peng et al. 2023). Based on the waste sources, research has demonstrated that industrial waste is the primary source of waste (Gaur et al. 2020). This waste is mainly composed of volatile compounds, wastewater, slag, and scrap generated during industrial production, which contain many hazardous substances, such as heavy metals, organic pollutants, and radioactive materials, leading to severe environmental pollution (Patel et al. 2022; Tawfik et al. 2022). Additionally, organic waste is generated by agricultural, animal, and wastewater treatment waste and the food industry. This type of waste can be used for value-added purposes, composting, or sent to landfills (Ren et al. 2018). Organic waste from natural environments may also be hazardous, as it contains toxic substances, such as ammonia and chlorine, which can cause air, water, and soil pollution.

According to the waste states, waste can be divided into solid waste (Jha et al. 2022), hazardous waste (Jha et al. 2022), liquid waste (Mekonnen 2012), organic waste (Sharma et al. 2019), and recyclable waste (Ren et al. 2018). Solid waste is mainly generated from human activities such as manufacturing, agriculture, and mining, and the treatment methods include recycling, incineration, and landfill (Bhatt et al. 2018). Solid waste types include construction, household, industrial, hazardous, electronic, medical, and agricultural waste (Peng et al. 2023; Vyas et al. 2022). Hazardous waste contains toxic, flammable, combustible, radioactive, and corrosive waste, mostly from electronic and biomedical waste (Akpan and Olukanni 2020). Hexavalent chromium liquid, mercury liquid waste, corrosive and alkaline liquid waste, cyanide liquid waste, and heavy metal liquid waste are all examples of liquid waste. In particular, corrosive and alkaline liquid waste makes up 12.4%, organic liquid waste 32.2%, and heavy metal liquid waste 47.9% (Ho and Chen 2018). If appropriate control measures are not taken, most liquid waste is hazardous industrial waste, which can significantly negatively affect the environment and public health (Tong and Elimelech 2016). Recyclable waste is refuse that can be removed from the waste stream and used as a raw material to create new products like paper, glass bottles, and ceramics (Fenta 2017). Some refuse recycling techniques include biological re-treatment, energy recovery, and physical re-treatment (Waheeg et al. 2022).

To summarize, waste types mainly include solid waste, hazardous waste, liquid waste, organic waste, and recyclable waste. The main sources are individuals, industry, agriculture, and transportation. Treatment methods include recollection, incineration, landfill, biological, and pyrolysis.

## Artificial intelligence in waste management 

The utilization of artificial intelligence has the potential to bring about a revolution in municipal waste management by enhancing the effectiveness of waste collection, processing, and classification. Artificial intelligence-based technologies like intelligent garbage bins, classification robots, predictive models, and wireless detection enable the monitoring of waste bins, predict waste collection, and optimize the performance of waste processing facilities. The details are shown in Table 1. By leveraging artificial intelligence, municipalities bin reduce costs, improve safety, and reduce environmental impacts associated with waste management.

**Table 1:** Application of artificial intelligence to waste management. The main applications of artificial intelligence in waste management include intelligent garbage bins, garbage-sorting robots, and prediction models. Categorize and compare the sum of key information to the conclusions drawn

Type	Measure	Key information	Results/benefits	References
Smart garbage bin	Sensor network	1. Garbage bin monitoring 2. Collect data 3. Analyze information 4. Road planning	Used to collect municipal waste	Khan et al. (2021); Muyunda and Ibrahim (2017); Neetha et al. (2017)
	Ultrasonic sensors	1. Garbage will not overflow 2. The lid will open automatically 3. Automatic detection of garbage levels	Digital garbage bin	Praveen et al. (2020a); Wijaya et al. (2017)
	Ultrasonic sensors Internet of things	1. Garbage separation 2. Connect to the internet 3. Garbage level monitoring	Garbage bin network	Karnalim et al. (2020); Saranya et al. (2020); Mustafa and Azir (2017)
	Ultrasonic sensors Red external sensor	1. Identify garbage 2. Move straight lines 3. Garbage level monitoring	The garbage bin can be moved automatically in front of people	Pawar et al. (2018); Rajathi G et al. (2020)
Garbage-sorting robot	Height map Near-red extra-specular spectral image	1. Separate concrete, bricks/blocks, and mortar 2. Automatically grasp objects 3. Throw into the corresponding recycling area 4. Handle heavy objects without prior treatment	The sorting efficiency can reach 2028 selections/hour, and the accuracy of line identification is almost 100%	Kshirsagar et al. (2022); Xiao et al. (2020)
	Computer vision simultaneous localization and mapping	1. It can successfully avoid obstacles and carry out automatic patrol 2. Detect recyclables 3. Generate a three-dimensional circumferential map of robot positioning, navigation, and path planning	The applicability of recycling robots has been expanded, and the robustness has been improved	Feng et al. (2022); Wang et al. (2020)
	Deep learning simultaneous localization and mapping	Map reconstruction, navigation, repositioning, waste detection, and sequencing	The error difference is small	Bobulski and Kubanek (2021b); Chang et al. (2020); Chen et al. (2022b); Zhou et al. (2021)
	Computer vision Hyperspectral image	1. Adjust the gripper 2. No need for additional electrical gas or pneumatic connection to drive the gripper	Three pannings and angle grabs	Leveziel et al. (2022); Liu et al. (2021a); Yang et al. (2022b)
Predictive model for waste production	Predictor exclusivity Integrated empirical pattern decomposition	1. High-precision municipal solid waste prediction model was obtained 2. The predictor exclusivity and cross-prediction methods should be applied to the analysis of artificial neural network	Significantly improve the accuracy of large-scale forecasting	Ghanbari et al. (2023); Wu et al. (2020)
	Random forest algorithm Support vector machines	A radio frequency model is proposed that can improve prediction performance using small classification datasets	Use small categorical datasets to improve prediction performance	Abbasi and El Hanandeh (2016); Adnan et al. (2017); Cha et al. (2021); Cha et al. (2020)
	Principal component analysis	1. Convert category variables into continuous variables 2. Predictive performance has been improved	The average information on the waste generation rate of the observed values was 1165.04 kg/square meter, and the predicted value was 1161.52 kg/square meter	Cha et al. (2023); Cha et al. (2022); Minoglou and Komilis (2018)
	Gradient enhancement regression model	A gradient-enhanced regression model was developed to predict weekly waste production	Presents waste production trends in New York and collects comprehensive data	Johnson et al. (2017); Sunayana et al. (2021)

### Smart bin systems

Conventional garbage bins solely collect waste, and sanitation workers must carry out manual inspections to assess the trash level in the bins. This approach is not efficient for routine waste disposal inspections. Moreover, due to the frequent filling of the containers, disease-causing organisms and insects tend to breed on them (Noiki et al. 2021). Therefore, designing intelligent garbage bin monitoring systems to manage garbage is essential in constructing smart cities.

Numerous research studies on intelligent garbage bins have focused on two key functions: automatic waste classification and monitoring. These studies offer a potential solution for cities to achieve an effective garbage collection system. An intelligent garbage bin can be created by utilizing a system on a chip produced by the Espressif systems (ESP 8266) module, automatically detecting objects and setting thresholds within the bin. The information gathered can then be transmitted to another node for further analysis and processing (Praveen et al. 2020b). For example, Praveen et al. (2020a) designed a garbage bin with two main pins: the trigger pin connected to the sensor and the echo pin. An ultrasonic sensor is placed at the top and bottom of the cover. Rajathi G et al. (2020) designed a robot garbage bin with two sensors installed at the bottom, which moves along a straight line. An obstacle sensor is embedded on one side of it, which can sense black and emit a buzzer sound to indicate that the garbage has stopped storing for some time. In addition, an ultrasonic sensor can be placed at the bin’s edge to detect the waste level (Mbom et al. 2022). The status of the container will be updated on the web page via the wireless fidelity module, showing whether it is full or empty. Some researchers design bins that separate and monitor garbage using Arduino and wireless fidelity (Samann 2017). It has an automatic metal and non-metal separator. Using NodeMCU, the bin’s water level can be monitored in real time and sent to the cloud for further analysis and processing (Saranya et al. 2020).

In summary, the research on smart garbage bins mainly focuses on automatically monitoring the garbage filling level and notifying users in time. The information is primarily received by sensors and transmitted through the network. Intelligent bin systems can potentially increase the efficiency of garbage collection, reduce the spread of diseases, and enhance the city’s overall environment. However, the cost of implementing smart garbage bins is relatively high, making it challenging to promote them widely. To address this issue, the government could consider funding policies to reduce the cost of smart garbage bins, making them more accessible to the general public. Furthermore, the regular operation of these bins can be affected by environmental factors such as temperature and humidity. Thus, dedicated personnel must regularly check and maintain the garbage bins. Therefore, it is crucial to focus on developing and promoting smart garbage bins in the future.

### Waste-sorting robots

Garbage classification is strongly recommended for municipal solid waste management, and using robots can substantially enhance the efficiency of garbage classification. However, robots require advanced visual and operational skills to function in highly heterogeneous, complex, and unpredictable industrial environments for garbage classification (Koskinopoulou et al. 2021). Recent research has focused on improving the accuracy and efficiency of garbage classification robots, which requires the development of better sensors and cameras to identify different types of waste, as well as improved artificial intelligence algorithms for classifying waste. Utilizing hyperspectral images to locate the target region of interest is a promising approach (Xiao et al. 2020). Based on previous research, robots can cope with complex field conditions by adding simultaneous localization and mapping technology and instance segmentation methods. They can automatically collect construction and demolition waste (Wang et al. 2020). Deep learning technology, such as instance segmentation, can accurately detect the contours of all objects in an image, including construction and demolition waste (Chen et al. 2022b). Given the complexity of construction sites and the large amounts of construction waste generated, manual collection and classification are often inefficient and pose safety risks. Consequently, the recovery of construction waste has become a research focus. (Yang et al. 2023b).

Researchers are currently investigating methods of integrating waste-sorting robots into existing waste management systems, such as utilizing robots to sort waste before it is sent to landfills. In this regard, studies have suggested a parallel robot model, with the primary concept revolving around a gripper that is fully integrated into a 4-degree-of-freedom similar robot structure (Leveziel et al. 2022). Researchers are also exploring using visual sensors to improve the performance of waste-sorting robots. For instance, a waste-sorting robot has been developed using deep learning techniques and optical sensors that can accurately identify and classify different types of waste (Mao et al. 2022).

In conclusion, trash-sorting robots have the potential to significantly increase waste management effectiveness, decrease labor expenses, and boost refuse classification precision. However, some argue that waste-sorting robots are impractical due to their high installation and maintenance costs compared to traditional waste-sorting methods. Nonetheless, researchers are exploring more affordable ways of creating waste-sorting robots, such as utilizing less expensive materials or designing robots operating in diverse settings. Additionally, efforts are being made to improve the robot’s structure, sensors, waste classification algorithms, and robotic arms to make them more effective and efficient. Waste-sorting machines will continue to be of great interest and play a significant part in real-world uses in the future.

### Sensor-based waste monitoring

**Fig. 2:**
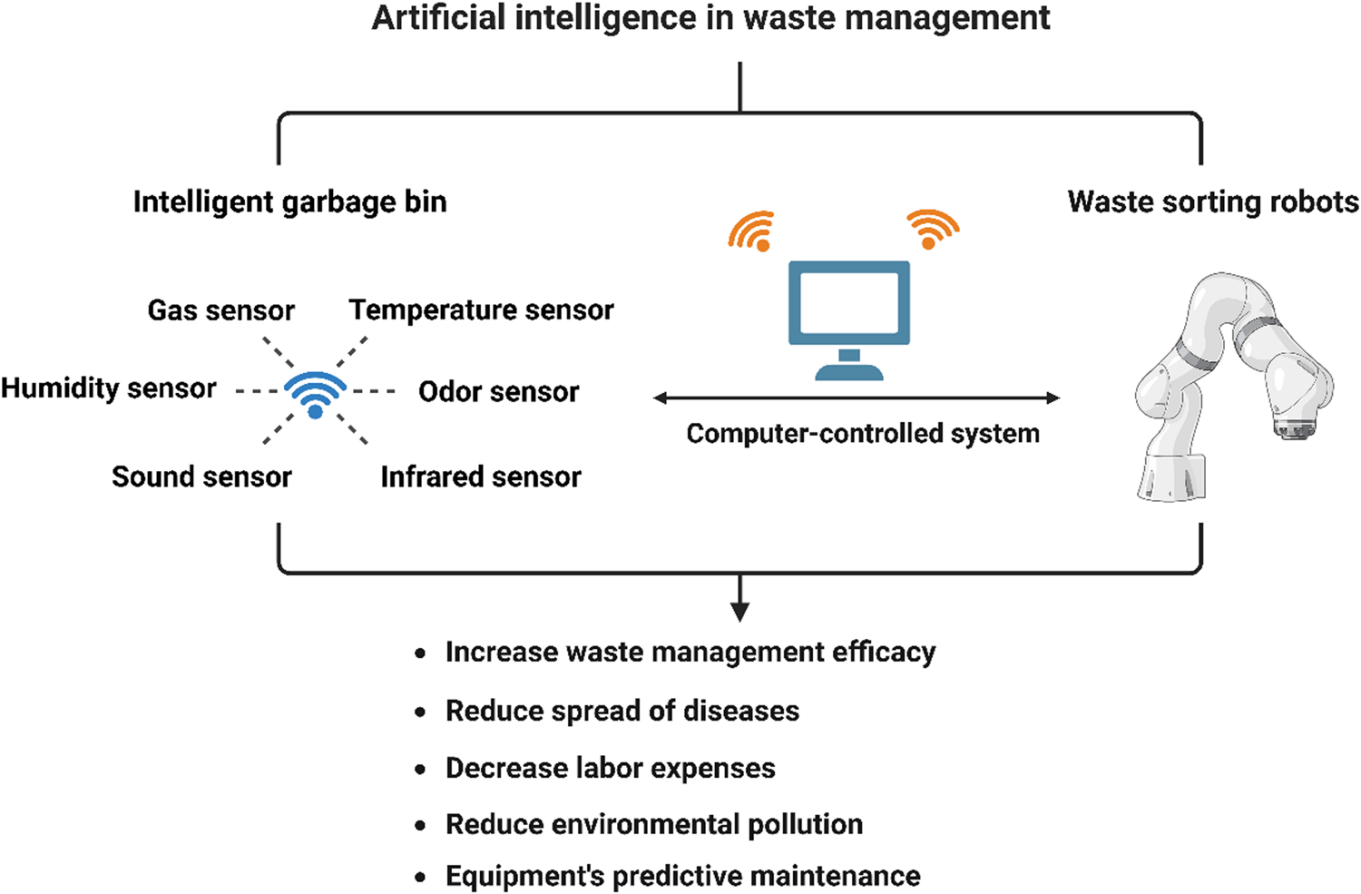
Uses of artificial intelligence in the garbage bin and waste robotic sorting. These include real-time garbage bin monitoring to optimize waste collection routes and prevent bin overflows. Additionally, intelligent garbage sorting can improve recycling efficiency and reduce contamination. In contrast, robotic waste sorting can utilize robotic arms to sort waste in recycling facilities, increasing the speed and accuracy of sorting while reducing the need for manual labor. Artificial intelligence can also be used for predictive maintenance to anticipate when waste-sorting equipment will require maintenance, reducing downtime and extending equipment lifespans. Lastly, waste management optimization using artificial intelligence can consider factors such as traffic, weather, and population density to enhance the efficiency of waste collection and processing

Sensor-based waste monitoring is a technology that utilizes sensors to track the amount of waste generated, identify the sources of waste, and measure the effectiveness of waste management strategies in a specific area. Wireless sensor network is a network composed of many self-organized wireless sensors installed in the network to monitor the physical or environmental parameters of the system (Gurram et al. 2022). As illustrated in Fig. 2, a typical wireless sensor network architecture for solid waste treatment systems includes various sensors, such as temperature, humidity, odor, infrared, gas, and sound sensors. Increase waste management efficacy.

These sensors can be used to monitor parameters in real time, thus better controlling the waste treatment process. For instance, an electronic nose can be developed using sensors to quantify odor concentration in real time to help treat wastewater (Burgués et al. 2021). Additionally, Sivaprakasam et al. (2020) proposed using a non-contact microwave sensor for in situ process monitoring of nuclear waste glass melts in cold crucible induction melting furnaces. In addition, they designed infrared sensors to determine the filling level of the carriages, gas sensors to detect hazardous gases, temperature, humidity sensors, and sound sensors to monitor noise pollution.

An intelligent garbage bin monitoring system based on a Zigbee network structure was suggested by Karthikeyan et al. (2017), in which the terminal nodes installed on the garbage bins detect the level of unfilled. Raaju et al. (2019) suggested using a solar energy collection device to power the wireless sensor network to increase the nodes’ lifespan. The major drawback of this system is its inability to display real-time filling levels of the garbage bin. Jino Ramson et al. (2017) suggested a solar-powered wireless monitoring unit with a sensor to measure the level of the can when it is empty and transmits the information to solar-powered wireless monitoring unit to address this problem. A self-powered, direct-connection wireless sensor network solid waste management system will be created by sending the data collected from numerous sensor nodes to the central monitoring station for analysis and visualization (Ramson et al. 2021). Additionally, a progressive bar is created in the graphical user interface to symbolize the garbage bin’s dynamic unfilled level.

**Fig. 3:**
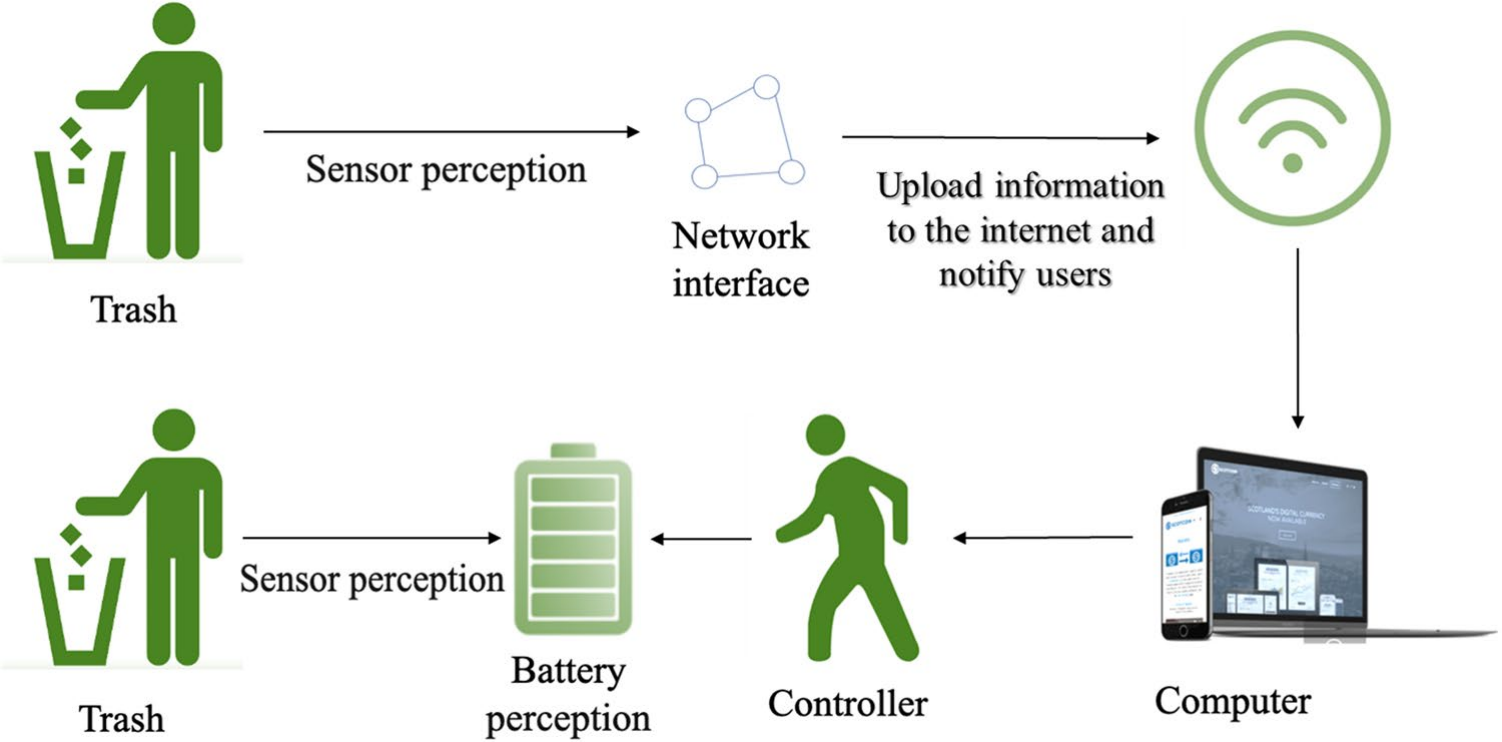
A typical wireless sensor network structure for a solid waste management system. A sensor is installed on the garbage bin. When garbage enters, the sensor can obtain information such as smell, weight, and humidity to classify the trash. At the same time, it can detect the environment of garbage bins and monitor the filling level of garbage bins. Users can monitor the status of garbage bins on the platform in real time as the information is uploaded through the internet

To summarize, the development of wireless sensor networks has been rapid, and there have been numerous studies on the application of sensors in waste monitoring, mainly through monitoring the level of garbage and then utilizing the network to notify users (Joshi et al. 2022). Many studies have reported applying different machine learning-based methods in waste management to predict and optimize municipal solid waste generation, detection, collection, classification, and properties. The mechanism is shown in Fig. 3.

### Models to predict waste generation

Research on waste generation prediction models has recently gained increasing attention, and various models have been proposed to predict better the amount of waste generated (Kolekar et al. 2016). These models include statistical, machine learning, deep learning, and fuzzy models. Artificial intelligence algorithms are considered the most advanced models for reliable waste generation prediction, as they possess unique capabilities (e.g., data input, learning, and prediction) (Coskuner et al. 2020).

Artificial neural networks are one of the nonlinear models widely used for modeling various urban waste management processes due to their robustness, fault tolerance, and ability to describe the complex relationships between variables in multi-variable systems (Cha et al. 2020). Machine learning algorithms such as artificial neural networks multilayer perceptron, support vector regression algorithms, linear regression algorithms, decision tree algorithms, and genetic algorithms can be used to develop models with better predictive performance on small datasets composed mainly of categorical variables (Cha et al. 2022, 2017; Golbaz et al. 2019). For cities with historical records, past historical data can be referred to and integrated with multiple datasets to establish a gradient boosting regression model. For example, Johnson et al. (2017) developed a short-term prediction model for garbage generation in New York, achieving an average accuracy of 88%. To analyze urban waste prediction models in cities with limited historical data, a method that includes all predictive factors can be utilized to evaluate regional differences and their influence on waste prediction. By assessing the model’s dependence on predictive factors, the impact of regional differences on urban waste prediction can be analyzed (Wu et al. 2020).

In conclusion, due to the rapid development of artificial intelligence, it has been widely used in waste generation prediction models. Artificial intelligence systems commonly used in waste management include artificial neural networks, support vector regression, linear regression, decision trees, and genetic algorithms. Among these, artificial neural networks have been widely implemented in waste generation prediction applications, followed by support vector machines.

### Monitoring and tracking waste materials 

Artificial intelligence technologies can be used to facilitate more efficient and effective waste classification and recycling. Machine learning techniques can be employed to identify the type of waste, such as plastics, metals, paper, and other materials, for more accurate and efficient recycling (Chen 2022). Artificial intelligence-based systems can also monitor the recycling process for anomalies, such as incorrect material classification or material contamination, and alert the relevant personnel to take corrective measures.

Furthermore, artificial intelligence can optimize the recycling process by analyzing the data from the recycling process and suggesting improvements (Pouyanfar et al. 2022). Additionally, artificial intelligence can be essential in measuring and tracking waste (Ponis et al. 2023). This can help to ensure that materials are recycled most efficiently and effectively.

Artificial intelligence can significantly improve the efficiency of environmental pollution information acquisition (Liu et al. 2021b). With the development of big data technology, the application of artificial intelligence can quickly improve the efficiency of information acquisition. Artificial intelligence has powerful perception capabilities, which can more effectively identify the source of environmental information and make basic judgments on the current environmental situation. For example, artificial intelligence can identify the location and size of noise pollution sources through sound recognition and present the noise situation in the area through spectrum analysis, so decision-makers can intuitively understand the noise distribution (Pan et al. 2018). Moreover, artificial intelligence can also achieve good environmental management (Zhu et al. 2022). By installing intelligent terminals in each garbage bin, various state information can be obtained in real time and further analyzed and processed. For example, it can detect whether the garbage bin is complete and use gas sensors to classify it into recyclable and non-recyclable categories (Rabano et al. 2018). This can reduce labor costs and improve classification efficiency, thus achieving good environmental management.

The utilization of artificial intelligence in waste recycling has gained widespread application. This includes optimizing waste collection truck routes, identifying waste management facilities, simulating the waste transformation process, and integrating various technologies such as radio frequency identification (Zhou and Piramuthu 2013), a global positioning system (Hidalgo-Crespo et al. 2022), and geographic information system (Zewdie and Yeshanew 2023) to monitor solid waste collection trucks and containers. Moreover, machine learning and image processing techniques have been combined with these technologies to automatically detect the level of containers (Vitorino de Souza Melaré et al. 2017). However, further research is still needed to explore integrating remote sensing technologies and artificial intelligence. On the one hand, it is necessary to use remote sensing technologies to quickly update the data of domestic waste and realize the dynamic planning of urban household waste management with a business system that can be continuously updated and run for a long time. In addition, it is crucial to further incorporate the expertise accumulated by specialists in this field into the system to achieve intelligent decision support for garbage management.

In summary, artificial intelligence’s rapid advancement has enabled its waste monitoring application to become a new research direction. Developing artificial intelligence platforms for waste monitoring is a highly sought-after research topic. The artificial intelligence platform receives data collected by monitors and sensors, which is then transmitted to the artificial intelligence server. The data train, optimize, and predict on the platform, generating intelligent waste management prediction models. These models can improve the quality and efficiency of pollution tracing and environmental problem-solving, providing effective solutions to environmental challenges.

## Chemical analysis of waste using artificial intelligence

Recent applications of machine learning have attracted considerable interest in areas including waste-to-energy conversion (Ahmad et al. 2015), biochar for metal and organic compound adsorption (Ascher et al. 2022; Dubdub and Al-Yaari 2021), municipal solid waste treatment (Goutam Mukherjee et al. 2021), and the oxidation of micropollutants (Ascher et al. 2022). Table 2 summarizes the latest applications of artificial intelligence in waste chemistry.

**Table 2:** Application of artificial intelligence in waste chemistry. This table summarizes 13 articles and divides the application of artificial intelligence in waste chemistry into three parts, mainly waste pyrolysis, carbon emission prediction, and energy conversion. Key information is also summarized

Field	Key information	Reference
Waste pyrolysis	Linking inputs to corresponding responses can improve machine learning by understanding the mathematical relationships between complex processes and algorithms. The study includes input variables such as feed capacity (kg/h), pyrolysis temperature (°C), and steam residence time (s). Raw material traits, including the composition of different plastic types, final analysis, and particle size (mm), are also considered input factors in the research	Ascher et al. (2022); Cheng et al. (2020); Mutlu and Yucel (2018); Yaka et al. (2022)
	These models can identify and evaluate catalysts that optimize hydrogen generation while minimizing carbon dioxide yield. Additionally, these models can be utilized to optimize hetero-catalyst loading during hydrothermal gasification and replicate the sodium hydroxide-catalyzed hydrothermal gasification of waste biomass to investigate the environmental impact of the process	
Estimate carbon emissions	The makeup of the carbon source (biochar, fossil, and inert) is essential in deciding greenhouse gas emissions from solid waste burning. Machine learning techniques, such as random forests and support vector machines, can uncover latent connections and forecast the properties of solid refuse groups. The carbon content of biological sources and fossils can be calculated in terms of mass using infrared spectroscopy and machine learning, allowing researchers to evaluate the effect of solid refuse burning on decreasing carbon emissions and saving substantial labor and reagents	Guo et al. (2021); Schwarzböck et al. (2018); Wang et al. (2021); Yuan et al. (2021)
	In biological processing, machine learning algorithms can be used to separate impurities from raw materials, compost, and solid digests. This can help reduce possible environmental risks and improve the profitability of compost and anaerobic digestion products	
Waste-to-bioenergy	Porous carbon produced from biomass refuse is a complex material extensively used in sustainable waste management and carbon capture. Biogas power generation is a form of sustainable energy fed by biological refuse produced by people and animals. It is part of the circular economy and is regarded as one of the most energy-efficient and ecologically beneficial bioenergy production technologies. However, biogas power production necessitates a lengthy response time and a complicated input, with no means to integrate machine learning and deep learning models	Chiu et al. (2022); Huang and Koroteev (2021); Kalhor and Ghandi (2019); Liu and Karimi (2020); Zaied et al. (2023)

### Prediction of pyrolysis conditions for plastic recycling

There is currently insufficient capability in the world’s waste management systems to securely dispose of or recycle all waste plastics, which inevitably increases the number of waste plastics dumped into the environment (Osman et al. 2023). Each year, the seas are thought to receive 8 million tonnes of microplastics and 1.5 million tonnes of main microplastics (Lau et al. 2020). For billions of years, waste polymers can deteriorate in the environment. Due to ineffective pre- and/or post-user management and widespread landfilling of refuse plastics, pyrolysis as a conversion technique can overcome severe ecological and environmental obstacles. Machine learning methods can be used to forecast the continuous and non-catalytic process products of refuse plastic pyrolysis.

Through data ingestion and experience, machine learning can instantly improve to correlate inputs to matching reactions and comprehend the mathematical relationships between intricate processes and algorithms (Ascher et al. 2022). Several algorithms have been recently reported to simulate pyrolysis or gasification processes. These include artificial neural networks, tree-based algorithms such as decision trees and random forests, and support vector machines (Mutlu and Yucel 2018; Yaka et al. 2022). Artificial neural network methods are the most popular machine learning techniques, for example, in the context of pyrolysis simulation by using tar, coke, and permanent gas interactions to model biomass gasification. Artificial neural network methods outperform realistic gas balance models that predict gasification products (Cheng et al. 2020). Tree-based methods and support vector machines have been successfully implemented in waste management. Cheng et al. (2020) proposed combining random forest-based predictive models with life cycle assessment and economic analysis to evaluate different pyrolysis feedstocks comprehensively. In addition, support vector machines have been widely used in pyrolysis prediction tasks. For example, support vector machines perform better than artificial neural networks at *R*^2^, and root means a squared error in predicting pyrolysis biochar yield (Cao et al. 2016).

The input factors of particle size (millimeters) allow for the classification of the composition of various plastic types, such as polyethylene, polypropylene, polystyrene, polyvinyl chloride, and polyethylene terephthalate, in the research (Osman et al. 2023). Additionally, the factors included the ashless chemical components of carbon, hydrogen, oxygen, nitrogen, and chlorine. Sulfur was not chosen, however, because it is typically insignificant compared to the elements mentioned above. As an additional reactor, working factors, feed capacity (kilograms/hour), pyrolysis temperature (Celsius), and steam residence time (seconds) were taken into account. Since they were only mentioned in a few carefully chosen sources, heating and carrier gas flow rates were not chosen as input factors. Where ranges were reported in the authorities, the mean values of the input variables were used in the study (Cheng et al. 2023).

In the hydrothermal gasification of waste biomass, recent research suggested a machine learning model for filtering and choosing catalysts. The writers used principal component analysis and split the dataset into three subcategories: non-catalysts, alkali metal catalysts, and transition metal catalysts (Li et al. 2021a). The created model yielded encouraging results when selecting and screening catalysts to maximize hydrogen production and decrease carbon dioxide production during hydrothermal gasification of discarded biomass. Comparable research used machine learning and a technology decision support system to optimize heterogeneous catalyst input during hydrothermal gasification (Gopirajan et al. 2021). The model demonstrated that catalyst addition had a favorable impact on hydrogen generation.

Artificial neural network techniques employing Levenberg–Marquardt and Bayesian regularization algorithms were utilized to analyze the environmental effects of waste biomass hydrothermal gasification with sodium hydroxide as a catalyst. Fózer et al. (2021) showed that a machine learning-based model could optimize and predict catalyst composition with a variance value of 0.965. Using sodium catalysts also increases the process’s ability to cause global warming. Future studies should focus on the effectiveness of catalysts in hydrothermal gasification processes and their effects on the atmosphere.

In conclusion, machine learning methods are often considered “black boxes,” making it challenging to apply them to study pyrolysis mechanisms and pathways comprehensively. Therefore, future research should integrate machine learning models with traditional modeling methods, such as kinetic studies, to provide more comprehensive information on reaction processes and routes. Some authors have suggested using feature alignment to evaluate the behavior and applicability of various input factors (Ascher et al. 2022). Additionally, future studies should clarify the opaque nature of machine learning algorithms to make them more accessible and facilitate the quick learning of the relationships between input and goal variables. To create more complete models, integrated predictive models should be developed to forecast critical aspects of relevant variables.

### Identifying modern and fossil carbon

Accurately measuring carbon emissions is essential to distinguish between the number of carbon sources in solid waste, including biogenic and fossil carbon. To this end, the three groups of carbon sources, namely the “biogenic carbon group,” “fossil carbon group,” and “inert carbon group,” are commonly used. Carbon dioxide outputs from paper, food refuse, and timber by the Bureau of Coast and Geodetic Survey are typically considered carbon neutral. However, carbon dioxide emissions from the Freight Classification Guide System are linked to climate change (Schwarzböck et al. 2018). Therefore, the findings may be overestimated if biomass-derived carbon emissions are not considered when calculating greenhouse gas emissions from solid refuse incineration. Hence, the Freight Classification Guide System and the Bureau of Coast and Geodetic Survey shares are crucial markers to evaluate how well solid refuse burning reduces carbon emissions. Machine learning, a tool for data extraction that identifies patterns, has shown promise in resolving challenging environmental issues (He et al. 2021). Machine learning algorithms such as random forests and support vector machines have become powerful tools for identifying hidden relationships that can predict the characteristics of different solid waste groups using established datasets and literature data. However, there are three main reasons why these methods may not be widely adopted: (1) limited data size and quality can hamper performance (Li et al. 2021b); (2) poor interpretability can make it challenging to extract relevant information (Visser et al. 2022); and (3) heavy computation requirements can result in extended processing times (Yan et al. 2021).

Moreover, many studies have focused on only one or, at most, two types of models, providing limited data for comparing the prediction accuracy of various models based on the same waste dataset (Wang et al. 2021). In the case of separately weighted garbage, the reduced total reflection infrared spectra to determine the mass-based amounts of biogenic and fossil carbon, Fourier-transform infrared, can be used with a machine learning method. This information can then be used to determine how solid refuse burning will affect the reduction of carbon emissions. This can save a lot of labor and chemicals while producing quick and precise results. From this viewing point, this strategy has much to offer regarding environmental and commercial fiscal benefits. By separating contaminants like plastics and stones from feedstock, compost, and solid digestate, the machine learning algorithm can be used in bioprocessing to lower possible environmental risks and enhance the profitability of composting and anaerobic digestion products (Guo et al. 2021).

In summary, accurately measuring carbon emissions from solid waste requires identifying the sources of biogenic and fossil carbon. Machine learning algorithms such as random forests and support vector machines can be used to extract data to identify carbon sources. Furthermore, combining Fourier-transform infrared spectroscopy with machine learning methods can determine the quantity of biogenic and fossil carbon. This approach can save resources and produce fast and accurate results.

### Waste to energy 

Biogas for electricity generation is a renewable energy source that takes its input from organic waste produced by humans and animals and is part of a circular economy (Farghali et al. 2022; Salguero-Puerta et al. 2019). According to the International Energy Agency, this century will see a two- to threefold rise in energy consumption, using up many resources. According to data from 2020, the global energy supply was 584,523,552 exajoules. Of this supply, 29.47% was derived from oil, 26.80% from coal, 23.68% from natural gas, 9.84% from biofuels, and 5.21% from renewable sources (Farghali et al. 2023). Encouraging renewable energy to generate power is one of the primary growth areas. Among the various methods of energy production, biogas power generation is considered one of the most energy-efficient and ecologically favorable bioenergy production technologies. Biogas resources have high utilization rates, especially in the circular economy. For example, in Europe, biogas energy production has exceeded 6 million tonnes of oil, with a yearly growth rate of over 20% (Chiu et al. 2022). However, producing biogas needs complicated inputs and slow reaction times. Although researchers have recently begun using predictive models to analyze the input factors for biogas production, key factors have not been adequately analyzed, resulting in inconsistent output. To maximize biogas output, machine learning was incorporated to analyze and identify the key variables that significantly influence results, obviating the need for intricate computations and lowering the risk of mistakes (Farghali et al. 2023; Pence et al. 2023).

**Fig. 4:**
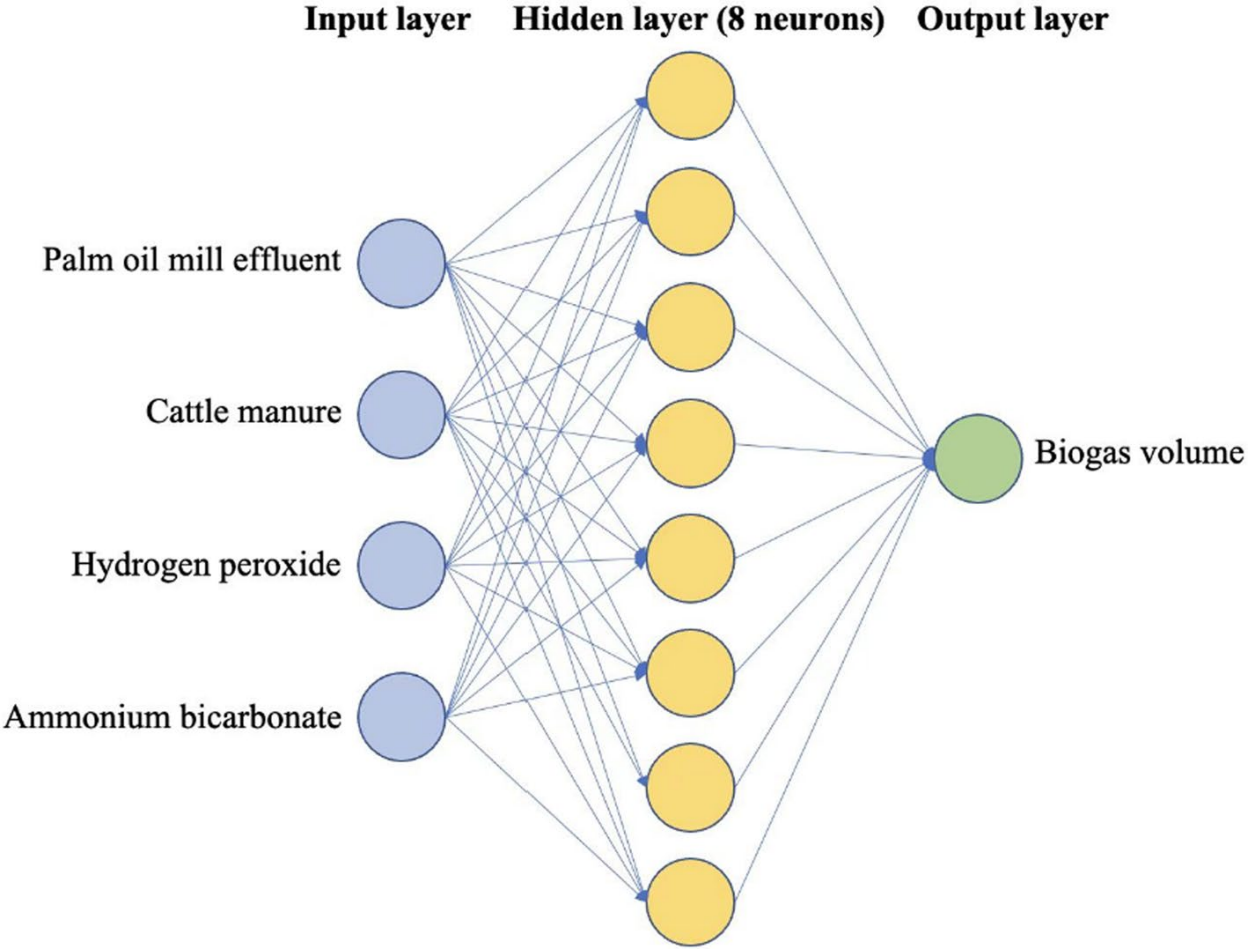
A neural network for predicting biogas volume using four input attributes. The diagram has eight hidden layers and one output layer to induce biogas prediction. It consists of three layers: input, hidden, and output

Currently, there are limited approaches that combine deep learning, machine learning, and neural networks for producing methane, as illustrated in Fig. 4. Some ongoing research is focused on developing a deep learning model for predicting faults in internal combustion engine power production, which can aid biogas plants in adjusting their maintenance and troubleshooting plans (Liu and Karimi 2020). Additionally, several studies have worked to develop an artificial neural networks model to forecast the optimum biogas output using particular sources like sugarcane bagasse and bovine manure (Ghatak and Ghatak 2018) and anaerobic co-digestion of palm mill wastewater (Zaied et al. 2023). Biogas generation studies and study findings use deep learning to analyze important waste and output forecasts compared to other renewable energy sources. Studies have looked into and made predictions about refuse gathering (Huang and Koroteev 2021) and some on output prediction, but waste inputs and outputs are rarely combined. Previous research focused on predictive models using machine learning techniques like artificial neural networks, *k*-nearest neighbors, and support vector machines. However, the effect of time on capability has not been considered. To make predictions, this research uses a time series model. According to a literature study, extended short-term memory deep neural networks can handle numerous variables, which makes them helpful for resolving issues with time series forecasting (Bouktif et al. 2018).

In conclusion, methane gas power generation is a renewable energy source whose input comes from organic waste produced by humans and animals and is part of the circular economy. Machine learning and deep learning models are used to analyze and identify key variables that significantly affect methane output. Extended short-term memory deep neural networks are used to predict waste input and output.

## Logistics, transportation and recycling

Logistics and transportation of refuse are essential components of waste management. Moreover, the waste’s logistics and transportation system is a critical hub connecting the waste source and treatment (Xia et al. 2022). However, the current waste logistics and transportation systems suffer from several shortcomings. Firstly, the costs associated with waste logistics and transport are prohibitively high, particularly during the collection phase. According to Sulemana et al. (2018), transportation costs incurred during waste collection account for approximately 70–80% of the total waste management costs. Secondly, personnel constraints lead to inefficiencies, such as disorganized collection plans and inadequate vehicles (Andeobu et al. 2022). Therefore, solutions based on artificial intelligence have been developed and implemented to optimize waste logistics and transportation processes (Abdallah et al. 2020). This involves optimizing waste transportation and logistics from four perspectives: transportation distance, transportation cost, transportation time, and efficiency.

Akdaş et al. (2021) introduced a method for vehicle routing using an ant colony optimization algorithm. First, collect 110 points of a particular city in Turkey. Then, convert the coordinates of the points and import them into the database. Visualize these points on a map and create a distance matrix. Finally, the ant colony optimization algorithm determines the shortest route on the distance matrix. Researchers found that the 10th iteration of the ant colony optimization algorithm can reduce the transportation distance by 13% to reach the optimal solution. In addition, other studies have shown that using Dijkstra and Tabu search algorithms can also reduce the distance of waste transportation (Rızvanoğlu et al. 2019). First, it used the Dijkstra algorithm to calculate the shortest distance between two coordinates. The Tabu search algorithm is to determine the fastest path between two coordinates. In the subsequent experiment, there were 200 waste collection points in a certain turkey area, 16,106 m. The two cases of transportation distance and 80 waste collection points, 5497 m transportation distance, are optimized. Rızvanoğlu et al. (2019) confirmed that Dijkstra and Tabu search algorithms could reduce transportation distance by 28%. In general, upon implementing the ant colony optimization algorithm, waste transport distances were reduced by 13% on average. Additionally, utilizing the Dijkstra-Tabu search algorithm reduced the waste transport distance by 28%. 

Babaee Tirkolaee et al. (2019) presented the simulated annealing algorithm for generating initial values based on a random algorithm. The simulated annealing algorithm is used for optimization based on obtaining the initial value. An area in Iran with 330 square kilometers and 43 recycling nodes is optimized using the simulated annealing algorithm (Babaee Tirkolaee et al. 2019). The simulated annealing algorithm reduced the total cost by 13.3%.

Amal et al. (2018) introduced genetic algorithms for optimizing vehicle routing. First, use a geographic information system to get the solution, including the vehicle’s route, subsequently utilizing the genetic algorithm to optimize the vehicle’s route. Finally, use ArcGIS and Python scripts to represent the optimal solution. Later, in an experiment in a city in Tunisia, after ten iterations of the genetic algorithm, the running time was reduced from 15.2 to 10.91 h. After optimizing the genetic algorithm, the running time of the vehicle was reduced by 28.22% (Amal et al. 2018). In addition, parallel annealing algorithms are also used to optimize vehicle collection paths (Zhang et al. 2020). Using the parallel annealing algorithm to optimize the waste collection path in Xuanwu District, Beijing, Zhang et al. (2020) found that the optimized scheme of the parallel annealing algorithm can reduce the time by 12% compared with the original scheme. However, this research also has certain limitations. For example, the collection of vehicle speed is a fixed value, which is impossible. Overall, the genetic and parallel annealing algorithms can reduce the transportation time by 28.22% and 12%, respectively. However, the optimization of the parallel annealing algorithm is limited by a fixed vehicle speed.

Akhtar et al. (2017) proposed an improved backtracking search algorithm on capable vehicle routing problems modeled under smart bins. Use the backtracking search algorithm to optimize based on the original route. At the same time, the data provided by the smart garbage bin is used to find the optimal range to reduce the number of garbage bins, thereby minimizing the distance. After four days of simulation experiments, Akhtar et al. (2017) found that the efficacy of waste collection increased by 36.78%. Algorithms and models can be developed further if more constraints and uncertainties are considered.

Furthermore, Nowakowski et al. (2020) proposed a harmony search algorithm for optimizing vehicle collection routes. After applying the harmony search algorithm to optimize vehicle routes for collecting electronic waste in a region of Poland, Nowakowski et al. (2020) found that the harmony search algorithm could increase the number of collection points visited by 5.4%. In summary, the backtracking search algorithm can increase efficiency by 36.78%, and the harmony search algorithm can increase the number of access points by 5.4%. Table 3 summarizes the optimization of artificial intelligence for waste logistics and transportation.

**Table 3:** Artificial intelligence optimization for waste logistics and transportation. The type of artificial intelligence refers to which artificial intelligence technology is used for optimization. The type of waste refers to what kind of waste is transported. Transportation distance, cost, and time reduction refer to the reduction of the transportation process after artificial intelligence optimization compared to before optimization. The increase in collection efficiency and the number of collection points refer to the increase in the transportation process after artificial intelligence optimization compared to before optimization. “–” stands for unmentioned

Types of artificial intelligence	Type of waste	Proportion of reduction in transport distance	Percentage of cost reduction	Percentage of time reduction	Increased ratio in garbage collection efficiency	Increased ratio in the number of collection points	References
Ant colony optimization algorithm	Solid waste	13%	–	–	–	–	Akdaş et al. (2021)
Dijkstra’s algorithm and Tabu search algorithm	Municipal waste	28%	–	–	–	–	Rızvanoğlu et al. (2019)
Simulated annealing algorithm	Municipal waste	–	13.35%	–	–	–	Babaee Tirkolaee et al. (2019)
Genetic algorithm	Solid waste	7.84%	–	28.22%	–	–	Amal et al. (2018)
Backtracking search algorithm	Municipal waste	36.8%	–	–	36.78%	–	Akhtar et al. (2017)
Harmony search algorithm	Electronic waste	–	–	–	–	5.4%	Nowakowski et al. (2020)
Parallel simulated annealing algorithm	Solid waste	–	–	12%	–	–	Zhang et al. (2020)

## Illegal dumping and waste disposal

Illegal dumping refers to disposing of waste and garbage in areas not designated for waste disposal, including private and public areas (Liu et al. 2017; Lu 2019; Niyobuhungiro and Schenck 2022). Illegal waste dumping can significantly impact the surrounding ecosystem, create social problems, and pose risks to human health (Lu 2019; Niyobuhungiro and Schenck 2021). With the rapid growth of the global economy, the quantity of waste generated annually is rising, and crimes leading to illegal dumping are also increasing (Du et al. 2021). Hence, governments worldwide must address the problem of illegal dumping as a critical waste management issue (Du et al. 2021; Niyobuhungiro and Schenck 2021). Identifying illegal dumping is an integral part of the process of dealing with illegal dumping. Municipalities have also adopted various methods to identify illegal dumping. For example, the South Korean government has installed cameras in areas where illegal dumping is concentrated to monitor and arranged for supervisors to patrol. However, installing cameras and arranging personnel to patrol requires many human and material resources, but it has not achieved good results (Kim and Cho 2022). As artificial intelligence technology advances, researchers have explored its potential to detect illegal dumping. The following section will discuss artificial intelligence’s use in identifying illegal dumping cases.

Shahab and Anjum (2022) used a multipath convolutional neural network model to identify and localize waste areas to identify illegal dumping. Because no data set for illegal dumping was identified in this model, Shahab and Anjum (2022) devised a quantitative investigation procedure. After training a multipath convolutional neural network model with 9000 pictures, the researchers used 3000 additional pictures to test the model’s classification accuracy, which was found to be 98.33%. Additionally, Thotapally (2022) proposed a method that utilizes the faster regions with a convolutional neural network features target detection framework and the residual network algorithm as a convolutional layer to identify street photos captured by surveillance cameras to determine the cleanliness of the street. Facts have proved that using the residual network algorithm as a convolutional layer method improves the accuracy of target detection and positioning. To sum up, both multipath convolutional neural network mode and residual network algorithm can judge whether there is waste dumping behavior through pictures. Among them, the accuracy rate of multipath convolutional neural network mode recognition is 98.33%.

Based on deep neural networks, Kim and Cho (2022) proposed a technique for monitoring illicit dumping that measures the distance between dumpers and garbage bags. The illegal dumping monitoring technology uses an openpose algorithm to identify the joints of the dumper and type of trash bag according to the “you only look once” (YOLO) model by detecting the distance between the dumper and trash bag to determine whether there is illegal dumping. After a series of experiments, the accuracy rate of the illegal dumping monitoring system is 93%. Additionally, since illegally dumped waste is often transported by trucks, Du (2020) suggested capturing images of waste-carrying trucks through monitoring systems and then using the YOLO algorithm to detect and determine whether they are involved in illegal dumping by comparing shape, color, and movement changes. In summary, the “you only look once” (YOLO) model algorithm can be used to distinguish whether there is an illegal dumping of humans and vehicles. The accuracy rate, when used to identify humans, is 93%.

Takahashi et al. (2022) used drones to collect river channel images and the faster regions with convolutional neural network features (faster r-convolutional neural network) model to train these images. Artificial intelligence can effectively identify garbage in pictures. However, using the faster r-convolutional neural network model to identify garbage in the city requires more training for a faster r-convolutional neural network model. Moreover, Youme et al. (2021) proposed using the single-shot detector algorithm (SSDA) to identify the pictures collected by drones. The SSDA can identify the location of garbage.

Nevertheless, the SSDA also has certain limitations. For example, only less litter can be identified in some covered regions such as wood areas. In addition, Padubidri et al. (2022) demonstrated using a basic convolutional neural network classification model and a residual block classification model to identify and report illegal dumping sites in high-resolution aerial imagery. With some limitations, recognition errors may occur and may not be suitable for recognizing low-resolution aerial images. In short, a faster r-convolutional neural network model and SSDA can identify garbage in the drone to shoot images. Deep learning models identify illegal leaning points in high-resolution aerial imagery. Nevertheless, there are some shortcomings in the above three methods.

Furthermore, Ulloa-Torrealba et al. (2023) introduced a method of illegal dumping detection using the random forest algorithm on the segmented high-resolution earth observation. However, this method has shortcomings, such as the inability to identify wastes smaller than 64 square centimeters and the high-resolution images not being real time. Torres and Fraternali (2021) presented residual network 50 and feature pyramid network to identify illegal dumps in aerial images. After testing, the method can reach 88% accuracy. Devesa and Brust (2021) proposed a method based on neural networks to identify illegal dumping sites in satellite images. However, this method has some disadvantages, such as the inability to identify small areas and obtain clear satellite images when there are clouds.

To sum up, the random forest algorithm, residual network 50, feature pyramid network, and neural network are all three methods that can be used to identify illegal dumping, but there are some shortcomings. Table 4 summarizes eleven methods for artificial intelligence to identify illegal dumping.

**Table 4:** Artificial intelligence models identify illegal dumping. Input data represent the picture recognized by artificial intelligence. Types of artificial intelligence stand for different artificial bits of intelligence that identify illegal dumping. Output result represents the result of artificial intelligence recognition. YOLO is the “you only look once” model algorithm

Input data	Types of artificial intelligence	Output result	Reference
Pictures with or without waste	Multipath convolutional neural network model	Determine if the vehicle is dumping garbage	Shahab and Anjum (2022)
Picture of vehicle	YOLO	Determine if the vehicle is dumping garbage	Du (2020)
Images from the drone	Faster regions with convolutional neural network features	Identify the location of garbage	Takahashi et al. (2022)
Pictures of the dump and the garbage	Openpose and YOLO	Determine if the dumper is dumping	Kim and Cho (2022)
Security camera shot of the street	Faster regions with convolutional neural network features	Determine if the street is clean	Thotapally (2022)
Images from the drone	Single-shot detector algorithm	Identify the location of the waste	Youme et al. (2021)
High-resolution aerial images	Basic convolutional neural network classification model	Identify illegal dumping sites	Padubidri et al. (2022)
High-resolution aerial images	Residual block classification model	Identify illegal dumping sites	Padubidri et al. (2022)
High-resolution earth observation	Random forest algorithm	Identify the location of the waste	Ulloa-Torrealba et al. (2023)
Aerial images	Residual network 50 and feature pyramid network	Identify illegal dumps	Torres and Fraternali (2021)
Satellite images	Neural networks	Identify illegal dumping sites	Devesa and Brust (2021)

Waste disposal reduces waste volume and accelerates waste purification through physical, biochemical, and pyrolysis gasification methods. Waste disposal methods can be categorized into four main types: waste recycling, waste incineration, waste composting, and waste landfilling (Chen 2022). Waste recycling involves collecting, treating, and reusing human-generated waste (Erkinay Ozdemir et al. 2021). Waste incineration uses high-temperature and high-pressure pyrolysis oxidation to reduce the volume of waste and eliminate hazardous materials (Chen et al. 2022a). Waste composting involves the controlled decomposition of organic matter in waste and its conversion into substrates and fertilizers (Aydın Temel et al. 2023; Wei et al. 2022). Waste landfilling involves filling waste into depressions or large pits, followed by anti-seepage, drainage, and air-guiding treatments.

The following sections will discuss using artificial intelligence in waste disposal concerning these four methods. The treatment and reuse of waste are two parts of waste recycling, and some researchers also focus on applying artificial intelligence in treatment and reuse.

Regarding waste treatment, Ziouzios et al. (2020) trained a convolutional neural network model to identify different wastes in waste recycling. The convolutional neural network model can classify wastes into five categories. After testing, the accuracy of the convolutional neural network model is as high as 96.57%. Next, the researchers will further experiment to improve the accuracy of the convolutional neural network model (Ziouzios et al. 2020). Moreover, the artificial intelligence technology using transfer learning realized the classification of 12 different models of smartphones (Abou Baker et al. 2021).

Regarding waste reuse, Qi et al. (2018) proposed a model for predicting strength that incorporated boosted regression tree and particle swarm optimization. The experimental results show that the strength prediction model can accurately predict the strength and reduce the required mechanical tests. In general, the convolutional neural network model and transfer learning can be applied to waste classification, while boosted regression trees and particle swarm optimization are applied to waste reuse.

Waste composting is widely used as a waste treatment method for organic matter. However, some problems in actual operation exist, such as maturity, heavy metals, and carbon dioxide emissions (Guo et al. 2022; Li et al. 2022; Sharma et al. 2021; Yang et al. 2023c). Therefore, artificial intelligence-based solutions are developed. Sharma et al. (2021) proposed using artificial neural network modeling to improve the maturity parameters of flower waste and cow manure in vermicomposting. After experiments, Sharma et al. (2021) found that the fertilizer optimized by artificial neural network modeling has enough nutrients to benefit the growth of plants.

Regarding risk control of heavy metals in livestock and poultry manure composting, researchers combined machine learning models such as layer perceptron regression and support vector regression to predict and optimize heavy metals in pig manure composting (Guo et al. 2022). After the experiment, Guo et al. (2022) found that the machine learning model and genetic algorithm can effectively reduce the risk of heavy metal pollution in livestock and poultry manure composting. In addition, Li et al. (2022) created a new machine learning model to predict carbon dioxide from green waste composting. A total of six different algorithms were used to predict carbon dioxide, with the random forest algorithm achieving the maximum prediction precision of 88%.

In summary, artificial neural networks can optimize compost maturity, and machine learning models can be used to predict heavy metals and carbon dioxide.

The waste landfill has become the most critical waste disposal method in most countries because of its large-scale operation and simple management (Mehrdad et al. 2021). However, landfills also present problems with siting, leachate, and odors (Abunama et al. 2019; Mohsin et al. 2022; Xu et al. 2022). Abunama et al. (2019) presented several models for forecasting leachate production rates in landfills. These models included single- and double-hidden layer artificial neural networks, multilinear perceptron, and support vector machine regression time series algorithms. After testing, Abunama et al. (2019) proved that the method of artificial neural network-multilinear perceptron with double hidden layers is optimal.

Meanwhile, regarding site selection for waste landfills, the fuzzy analytic hierarchy process–support vector machine and fuzzy analytic hierarchy process–random forest integrated models based on a geographic information system were developed Mohsin et al. (2022). Three landfills were selected to apply the model to siting landfills in a region of India. Xu et al. (2022) also constructed an artificial neural network model for ethanol, methyl sulfide, and dimethyl disulfide. They used a genetic algorithm to predict the odor emission rate of the landfill’s working face. Experiments show that the prediction accuracy is satisfactory. Overall, artificial neural networks can predict landfill leachate generation rates, vector machines and random forest models can be used for landfill siting, and genetic algorithms can be used to predict odor emission rates.

Although waste incineration is a common method of waste disposal, an improper operation can lead to adverse effects and problems. The complexity of waste incineration modeling arises from its nonlinear nature, strong coupling, significant delays, and high inertia. To overcome these difficulties, Chen et al. (2022a) developed an intelligent modeling approach based on deep learning models, which proved more accurate and effective in simulating waste incineration power plants. Wajda and Jaworski (2021) used an ant optimization algorithm after conducting laboratory experiments and practical tests, achieving satisfactory results. In addition, Cho et al. (2021) employed artificial neural networks to optimize the conditions of an incinerator, and their findings indicated that the model could reduce nitrogenous gas emissions by 34%. These optimization methods can help improve waste incineration processes’ efficiency and environmental impact.

## Identifying and recovering valuable resources

The rapid development of the global economy and urbanization has increased waste production, posing a serious problem for modern society (Chen et al. 2020). Governments mainly rely on landfill and waste incineration to manage waste, especially in developing countries, but improper disposal can cause environmental issues (Ferronato and Torretta 2019). However, the waste contains many recyclable materials, and recycling can reduce environmental impact and enable waste reuse (Zhang et al. 2021b). To promote waste recycling, many countries are implementing waste classification. However, manual classification is inefficient and prone to errors, hindering progress. Researchers are applying artificial intelligence to waste identification and classification to overcome these obstacles, proposing more reliable methods (Zhang et al. 2021b).

Zhang et al. (2021c) proposed a two-stage recognition–retrieval algorithm for waste classification. The first stage involves constructing a recognition model to sort waste into thirteen categories, and the second stage trains a recognition–retrieval model to classify waste into four categories. However, the algorithm has limitations, such as only identifying one waste type in mixed waste and low accuracy in classifying paper, tissue, and fabric. Moreover, Sousa et al. (2019) introduced a hierarchical deep learning method for sorting and identifying waste in food trays, which classifies waste into four categories based on material or ten categories based on shape using faster regions with convolutional neural network features. Similarly, Melinte et al. (2020) demonstrated a deep convolutional neural network that can classify municipal waste into five categories with an accuracy rate of 97.63% using single-shot detectors and faster regions with convolutional neural network features.

Based on the image classification model of deep learning, Zhang et al. (2021b) developed a method for adding a self-monitoring module to the residual network model. This model divides waste into six types according to the material. After experiments, it was found that the model’s accuracy was 95.87%. However, the model also has some limitations, such as the small amount of data in the data set, it is not realistic enough, and the actual situation in real life is quite different. Furthermore, Fahmi and Lubis (2022) developed a waste recognition system using a convolutional neural network to classify waste into inorganic and organic substances, with each group further subdivided into five subclasses. After conducting experiments, the waste recognition system achieved an average accuracy rate of 90%. Zhang et al. (2021a) proposed a waste identification and classification method using transfer learning and convolutional neural networks. The method categorized waste into five classes based on different materials, and the model’s classification accuracy was 82% through testing. However, the transfer learning and convolutional neural network models have some limitations, such as the simplicity of waste pictures used in the experiments and the gap between these pictures and real-life waste.

Shi et al. (2021) proposed a multilayer hybrid convolution neural network for waste classification, achieving an accuracy rate of 92.6% for classifying six waste categories based on material. Meanwhile, Na et al. (2022) employed image data augmentation and transfer learning to classify construction waste into five categories based on material. However, the model’s quality may be affected by increased data, and collecting pictures during sunrise and sunset should be avoided. Ziouzios et al. (2022) developed a waste detection and classification system using convolutional neural networks, achieving a 92.43% accuracy rate for classifying four waste categories based on material. However, the system has high hardware costs and energy consumption.

In order to better classify and identify textile waste, Du et al. (2022) have developed a deep learning model based on a convolutional neural network to better classify textile waste. The model can accurately classify textile waste into 13 categories based on material with a recognition time of fewer than two seconds and an accuracy rate of 95.4%. Bobulski and Kubanek (2021a) have demonstrated a deep learning-based classification system that uses a convolutional neural network to classify plastic waste into four categories based on different materials. Both factories and households can use the system. Toğaçar et al. (2020) have proposed a waste classification method based on a convolutional neural network that divides waste into recyclable and non-recyclable categories with a precision rate of 99.5%. Furthermore, Thumiki and Khandelwal (2022) have developed a real-time mobile application that uses image recognition and a convolutional neural network to classify waste into six categories based on material and determine whether it is recyclable or non-recyclable.

In summary, artificial intelligence, particularly deep learning models based on convolutional neural networks, has been shown to be effective in classifying waste according to their material and shape. These models can accurately identify and classify different types of waste, which can help with waste management and recycling efforts, as listed in Table 5.

**Table 5:** Artificial intelligence garbage classification and identification. The various types of artificial intelligence refer to different garbage classification and identification approaches. The classification index defines the type of classified waste, while the number of classifications indicates the number of categories the waste is divided into. The accuracy rate is the proportion of correct classifications to the total number of classifications made by artificial intelligence. If a value is not mentioned in the text, it is represented by a “–” symbol

Types of artificial intelligence	Classification index	Number of classifications	Accuracy rate	Reference
Recognition–retrieval model	Different types of garbage	Four	94.71%	Zhang et al. (2021c)
Faster regions with convolutional neural network features—material	Material	Four	72.8%	Sousa et al. (2019)
Faster regions with convolutional neural network features—shape	Shape	Ten	73.6%	Sousa et al. (2019)
Single-shot detectors	Material	Five	97.63%	Melinte et al. (2020)
Faster regions with convolutional neural network features	Material	Five	95.76%	Melinte et al. (2020)
Residual network model	Material	Five	95.87%	Zhang et al. (2021b)
Convolutional neural network	Organic and inorganic matters	Ten	90%	Fahmi and Lubis (2022)
Transfer learning and convolutional neural network	Material	Five	82%	Zhang et al. (2021a)
Multilayer hybrid convolution neural network	Material	Six	92.6%	Shi et al. (2021)
Image data augmentation and transfer learning	Material	Five	–	Na et al. (2022)
Convolutional neural networks	Material	Four	92.43%	Ziouzios et al. (2022)
Convolutional neural network and deep learning	Material	Thirteen	95.4%	Du et al. (2022)
Deep learning and convolutional neural network	Material	Four	–	Bobulski and Kubanek (2021a)
Convolutional neural network	Recyclable and non-recyclable	Two	99.95%	Toğaçar et al. (2020)
Image recognition and convolutional neural network	Material	Six	–	Thumiki and Khandelwal (2022)

## Improving public health and quality of life 

Implementing artificial intelligence technology to improve sustainable waste management can help reduce the use of natural resources without compromising the standard of living. This ensures a reduction in the generation of solid waste and its disposal to minimize its impact on health and the environment (Yigitcanlar et al. 2021; Yusoff 2018). The amount of solid waste generated worldwide is much greater than that of recyclables, and this trend is expected to continue (Kabirifar et al. 2021; Khudyakova and Lyaskovskaya 2021). By incorporating artificial intelligence technology for intelligent recycling, waste classification, and disposal in developed and developing countries, the municipal solid waste process can be strengthened, leading to more sustainable recycling methods (Tanveer et al. 2020).

To effectively manage solid waste generation, creating and implementing a strategic roadmap is important (Wath et al. 2010; Williams 2019). Additionally, accurately predicting the age of solid waste is crucial for achieving efficient municipal solid waste management, which can be accomplished through artificial intelligence (Yigitcanlar and Cugurullo 2020). Traditional waste-sorting techniques are being replaced by automated intelligent machines capable of multitasking and sorting large amounts of solid refuse. These machines are powered by artificial intelligence, can distinguish between different types of solid waste, and exhibit a high degree of autonomy in computer vision programs (Wirtz et al. 2019).

Urban waste is commonly known as “municipal waste,” and certain types of waste within this category require special handling and management due to their explosive, toxic, or polluting nature that poses a risk to public health and living conditions (Olugboja and Wang 2019). Municipal-level hazardous, toxic, or detrimental waste to the quality of life and living conditions must be managed precisely. This includes waste generated from operational processes such as combustion ash, raw sewage, toxic enzyme oil, waste material, scrap metal, asphalt waste, ceramic waste, slag, gravel, animal manure, animals, grains, ashes, artificial waste, materials used in urban waste management, menstrual waste, and the generation of menstrual waste, all of which have the potential to cause harm, toxicity, or infection to public health and the environment.

Intelligent bins exemplify how artificial intelligence is employed in municipal solid waste management. Waste management companies can utilize artificial intelligence technology to monitor garbage bins’ fill levels throughout a city. Municipalities and recycling companies can optimize their trash collection schedules, routes, and frequencies (Brynjolfsson and Mcafee 2017; Ortega-Fernández et al. 2020). This optimization reduces the time required to empty the bins while also reducing labor and fuel costs. Furthermore, artificial intelligence technology can detect when a bin is full and distinguish between various waste types. For example, smart bins can rapidly classify and sort garbage using machine learning algorithms (Yigitcanlar and Cugurullo 2020).

Artificial intelligence is critical in solid waste management by facilitating classification (Gundupalli et al. 2017). Artificial intelligence is used for intelligent classification by using cameras to automatically scan and analyze items on a conveyor belt using deep learning algorithms, similar to how it is used in manufacturing (Gundupalli et al. 2017; McKinnon et al. 2017). Recent studies have demonstrated that artificial intelligence-powered machines can process up to 160 recyclable materials per minute, compared to 30 to 40 materials per minute for human workers (Andeobu et al. 2022). Moreover, artificial intelligence-powered machines can operate continuously, highlighting deficiencies in classification and recycling facilities (McKinnon et al. 2017). Traditionally, solid waste management has been a labor-intensive process. However, thanks to advances in artificial intelligence, computer vision, robotics, and other cutting-edge technologies, municipalities can now improve public health and quality of life while reducing costs and eliminating the need for manual labor.

In summary, using artificial intelligence in waste management is becoming increasingly popular. An example is developing an artificial intelligence-based hybrid intelligent framework, which optimizes waste management and improves urban environment monitoring using graph theory and artificial intelligence technologies (Ihsanullah et al. 2022). By employing different approaches and algorithms based on artificial intelligence, this system can better accommodate various demographic groups, enhance environmental planning, and improve urban management’s efficiency, accuracy, and performance. Results show that the method improves the efficiency and accuracy of waste processing compared to other existing methods (Yu et al. 2021).

**Fig. 5:**
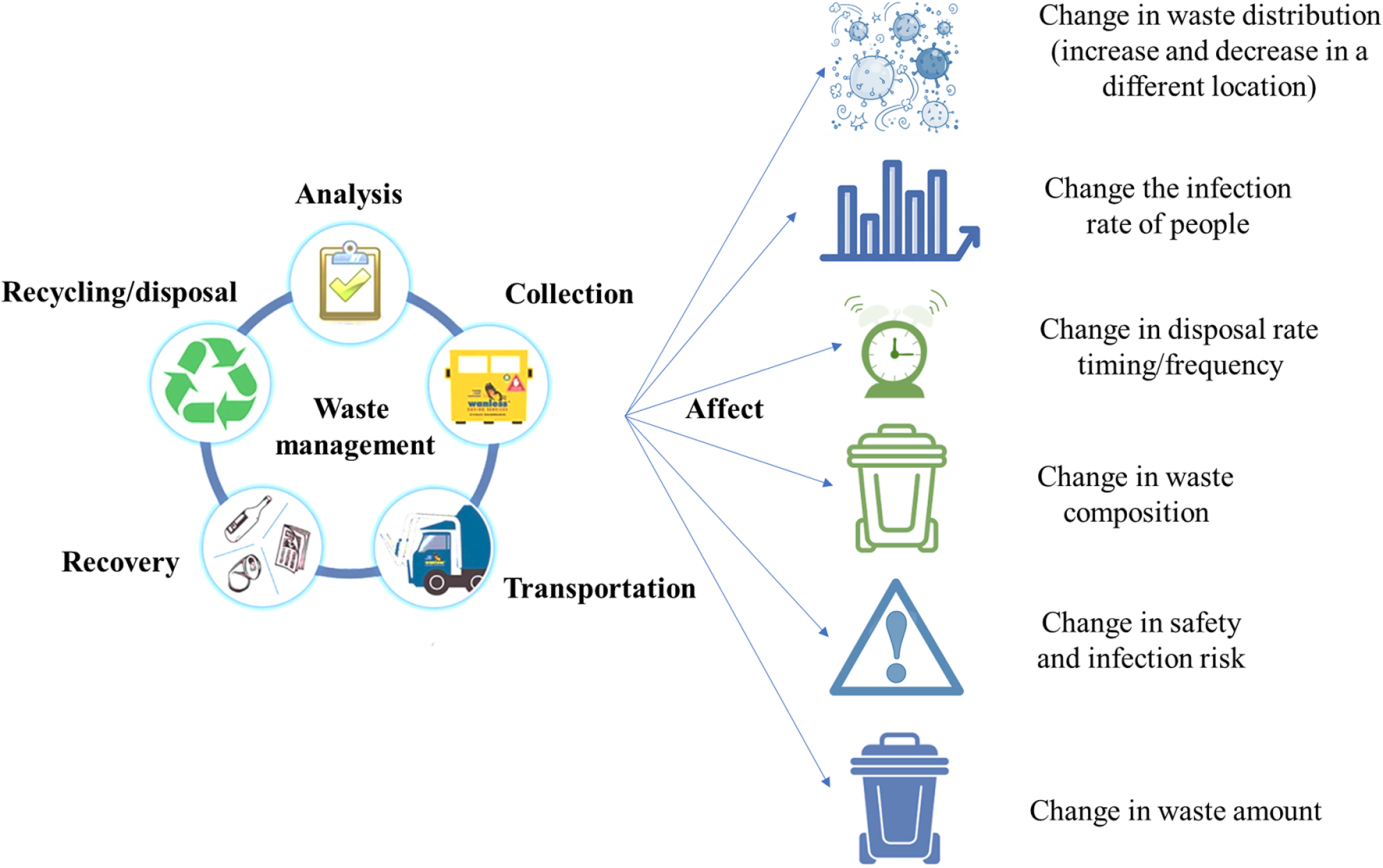
Impact of the coronavirus disease 2019 (COVID-19) on waste management. The COVID-19 pandemic has significantly affected the composition, timing, and frequency of waste disposal. It has also increased the risk of infection for the public due to the production of masks and medical waste that require manual handling. These changes in waste volumes have complex and interrelated impacts on municipal waste management, as depicted in the chart

Since the outbreak of the novel coronavirus (COVID-19) pandemic, the rapidly increasing amount of medical waste has posed more significant challenges to various regions’ disposal facilities and management capabilities. Medical waste has the characteristics of spatial pollution, acute infection, and latent infection, which can directly endanger human health. It can also cause severe consequences through soil pollution, water bodies, and the atmosphere (Yang et al. 2022a). As shown in Fig. 5, coronavirus disease 2019 (COVID-19) changes the composition of waste and the efficiency of waste disposal and increases the risk of infection in the population. Human intervention in solid waste management can be reduced through advanced intelligent waste management technologies, such as machine learning-based image classification and reliable item detection. Specific materials that improve ecological sustainability after a significant outbreak can be effectively recycled. These technological interventions will reduce the risk of human factor contamination in the waste management cycle, thus breaking the potential transmission chain of COVID-19 and similar viruses (Rubab et al. 2022).

The proposed medical waste monitoring and assistance system utilizes artificial intelligence to perform deep mining and analysis of personnel disposal behavior in the temporary storage of medical waste and working areas of medical and health institutions. The system can intelligently identify illegal and irregular behaviors on the scene and synchronously push alarm information to medical institutions and law enforcement personnel (Yu et al. 2021). The aim is to improve the management and disposal of medical waste, reduce the risks of infectious disease transmission and environmental pollution caused by medical waste, and ensure public health and safety (Lakshmi et al., 2015). By utilizing the technology of “internet + video surveillance and data tracking,” artificial intelligence can intelligently recognize illegal behaviors related to waste management without the need for the physical presence of law enforcement personnel. This can help reduce the risk of infection and transmission of diseases while also improving the overall efficiency of waste management supervision.

In summary, improper disposal of solid waste can adversely impact human health and the environment. However, current solid waste management systems struggle to keep up with the ever-increasing amount of waste produced globally (Popa et al. 2017). There are certain recyclable materials that some municipal solid waste and recycling services cannot process. The integration of artificial intelligence in solid waste management is a growing trend and has the potential to significantly improve sustainable waste management practices (Khudyakova and Lyaskovskaya 2021). Adopting artificial intelligence-driven automation in the management process of municipal solid waste will provide a sustainable approach to recycling and disposal (Popa et al. 2017). Thus, implementing these advancements in solid waste management can promote a healthier and better quality of life for all.

## Designing waste management systems for smart cities 

In the pursuit of creating environmentally friendly cities, waste management plays a critical role. Ensuring sustainable and livable urban areas requires improving solid waste management services and reducing waste, as hazardous solid waste can negatively impact air quality and soil safety (Herath and Mittal 2022). With the rise of smart cities, artificial intelligence technology in waste management has become more prevalent, primarily as a modeling and prediction tool for simulation and optimization. The most recent applications have primarily focused on modeling and optimizing solid waste generation, as well as predictive recycling processes, as demonstrated in Table 6.

**Table 6:** Artificial intelligence models and machine learning algorithms for solid waste prediction, reduction, and recycling. Algorithms differ in their information requirements for machine training, and their sizes vary across different algorithms. Each algorithm comes with its unique set of advantages

Input parameters	Output parameters	Types of artificial intelligence technology	Scale	Advantage	Reference
Vehicle journeys and the monthly volume of solid waste from the weight bridge	Estimation of the landfill’s size based on produced and collected solid refuse	Feed-forward back-propagation neural network	Medium term	Artificial neural networks learning methods are highly resistant to noise in a data set’s training data	Hoque and Rahman (2020)
Generation of municipal solid refuse in monthly time series	Generation of municipal solid waste	Support vector machines	Short term	Function well when there is a maximum marginal splitting of groups	Abbasi and El Hanandeh (2016)
Historical statistics on the weather, historical data on the amount of household waste collected, and historical tonnage data	Weekly municipal solid waste generation tonnage	Gradient boosting decision tree	Short term	It can be used for large data sets, and good for prediction problems	Johnson et al. (2017)
Individual building attributes, neighborhood socioeconomic characteristics, weather, and selected route levels are collected data	Construction-grade municipal solid waste generation	Gradient boosting decision tree	Medium term	The linear regression function demonstrated a higher correlation coefficient for the training data set than other models	Kontokosta et al. (2018)
Temperature, pH, stirring, and time of municipal waste-activated sludge pretreatment	Enzymatic activity	Multilayer perceptron network	Short term		Selvakumar and Sivashanmugam (2018)
Biomass sludge ratio, heating rate, and temperature	Percentage of massive losses	Multilayer perceptron network	Short term		Chen et al. (2017)
Human power, water, electricity, gas, and transportation	Biodepletion potential, acidification potential, and eight other environmental impact categories, as well as recycled materials	Multilayer perceptron network	Short term		Nabavi-Pelesaraei et al. (2017)
Tea waste volume, pH, polyacrylonitrile concentration, sample and eluate flow, eluate volume, and lotion concentration	Percentage of extraction of manganese and cobalt	Particle swarm neural network inverse	Short term	Sensitivity analysis can be performed	Nabavi-Pelesaraei et al. (2017)

Studies have suggested that integrating waste management into future smart cities with entire product lifecycles could be a potential step toward achieving “zero waste” (Lee et al. 2021). To reach this goal, three stages must be undertaken: Waste prevention, precise refuse collection, and functional value recovery from collected waste are all priorities (Yang et al. 2023a). Furthermore, the Internet of things waste management networks should be encouraged to improve the life cycle of products and their recycling value (Shukla and Hait 2022).

To effectively implement and manage waste models for smart cities, it is crucial to intelligently estimate solid waste generation. In this regard, artificial intelligence technologies such as artificial neural networks, support vector machines, decision trees, and adaptive neuro-fuzzy inference systems have been increasingly used due to their practical predictive capabilities for modeling the production of municipal solid waste (Ihsanullah et al. 2022). Artificial intelligence-based models in waste management research are commonly categorized by the forecast period duration: short term (days to months), medium term (up to 3–5 years), and long term (years in advance). Recent studies have demonstrated promising results in utilizing artificial intelligence with historical data, such as sociodemographic, economic, and management-focused data. Additionally, combining artificial intelligence with conventional waste management systems can be achieved by integrating the Internet of things technology (Ijemaru et al. 2022).

Artificial intelligence techniques have been used in multiple studies to predict specific types of solid waste generation, such as plastic waste (Kumar et al. 2018) and household packaging waste (Oliveira et al. 2019), and have proven to be efficient and feasible. However, analyzing and selecting key metrics is crucial to achieving accurate prediction performance and ensuring the dataset’s completeness and adequacy. In addition to waste forecasting, automated waste sorting and management are essential for better waste recycling. Advanced technologies, particularly the idea of smart cities, require this model. Artificial waste separation is inappropriate for smart cities, so smart waste classification models are typically multilayer convolutional deep learning models with some physical requirements. These requirements include a system with a conveyor belt, a pusher, and a garbage basket that will collect the garbage pushed by the hammer according to the waste category (Gondal et al. 2021). Therefore, artificial intelligence-based models can accurately forecast and evaluate solid waste generation and automate waste management and sorting for better recycling, which is crucial for cutting-edge technologies like smart cities.

In general, the techniques used in waste management applications fall into the following four categories (Zhang et al. 2012):

i. Space technology includes using global navigation and geographic information systems to track waste and manage waste collection and disposal.
ii. Identification techniques: such as radio frequency identification tags and bar codes which enable efficient waste tracking and monitoring
iii. Data acquisition technology, such as sensors and imaging, provides valuable waste generation and composition data, enabling better waste management decisions.
iv. Data communications technology, including wireless fidelity, Bluetooth, and global mobile communication systems, facilitates communication and data transfer between waste management systems and stakeholders

The steps to achieve “zero waste” include the following three main modules and components (Shukla and Hait 2022):

i. Establishing a framework for collating product life cycle data.
ii. Encouraging responsible citizenship through innovative waste reduction ideas.
iii. Developing an intelligent infrastructure with sensor-based technology for adequate garbage segregation, collection, and recycling.

In summary, smart cities have emerged as a global model that prioritizes sustainability. With the help of computing, networking, and data management advancements, institutions have successfully implemented efficient waste management systems that enhance the quality of life for citizens. However, the proposed model must be adapted to different demographics, waste types, and social needs and calibrated through real-world pilot studies. Extensive research into waste management in smart cities has produced numerous results.

## Process efficiency and cost savings

There is great potential to automatically detect waste in natural settings to improve waste management efficiency. For instance, Zhou et al. (2023) proposed a few-shot waste detection framework that employs faster regions with convolutional neural network features (faster r-convolutional neural network) to detect waste automatically in natural settings, thereby improving waste management efficiency. Their experiments revealed that this framework outperformed state-of-the-art detectors with 1.68% accuracy. However, using a faster r-convolutional neural network in the framework resulted in high computational complexity and slow operation speed. Moreover, Alqahtani et al. (2020) presented an urban waste management system that employs the cuckoo search algorithm to analyze waste sources, types, and vehicle capacity to optimize waste collection.

Experimental results have shown that waste recycling systems can improve waste management efficiency and collect waste within 15 min. Akhtar et al. (2017) proposed an enhanced backtracking search algorithm for optimizing waste collection routes based on the smart bin concept. The algorithm uses data from the smart garbage bins to identify the optimal range and reduce the number of garbage bins, thereby minimizing distance. After four days of simulation experiments, they reported a 36.78% increase in the efficacy of waste collection. These algorithms and models can be further developed by considering more constraints and uncertainties.

Shreyas Madhav et al. (2022) have developed a convolutional neural network-based recognition system for e-waste classification. The system can classify e-waste into eight categories with 96% accuracy, potentially leading to a 20% cost reduction within five years if implemented as a replacement for manual classification. To optimize waste collection, Internet of things (IoT) -based waste management software can collect relevant information, and the route of waste collection vehicles can be optimized using an ant colony optimization algorithm. Experiments have reported a 30% reduction in the direct cost of waste collection using this algorithm (Oralhan et al. 2017). Meanwhile, Babaee Tirkolaee et al. (2019) proposed a simulated annealing algorithm for generating initial values based on a random algorithm, which was then used for optimization. The algorithm was applied to an area in Iran with 330 square kilometers and 43 recycling nodes, reducing the total cost by 13.3%.

In summary, developing the convolutional neural network-based recognition system for e-waste classification can improve accuracy and reduce costs. Optimizing waste collection routes using algorithms such as ant colony optimization or simulated annealing can lead to significant cost savings.

Over the years, the use of artificial intelligence technology in various waste management fields has risen. However, with its increasing use, some potential challenges have also emerged. Among these is the black box problem of artificial intelligence, which arises due to the complexity of internal structures of most artificial intelligence models, relatively independent operation processes, and difficulty in estimating the relative significance of each variable, thereby limiting manual intervention (Ihsanullah et al. 2022). These black box problems can lead to uncertainty in applying artificial intelligence models (Guo et al. 2021).

The training and testing of artificial intelligence models, especially those that utilize deep learning and machine learning, require significant data. However, the waste management industry, particularly in developing countries, suffers from data scarcity and incomplete data, which hinder current research (Abdallah et al. 2020). Insufficient and outdated data can result in overfitting and reduced model training efficiency (Guo et al. 2021). One example is the deep neural network, which relies heavily on extensive testing and experimentation using large datasets (Ihsanullah et al. 2022).

Artificial intelligence has been increasingly implemented in waste management, but researchers often rely on preexisting models like faster regions with convolutional neural network features or convolutional neural networks. However, a lack of customized artificial intelligence models designed specifically for waste management exists. This requires collaboration between waste management and computational technology teams to develop tailored models (Abdallah et al. 2020).

**Fig. 6:**
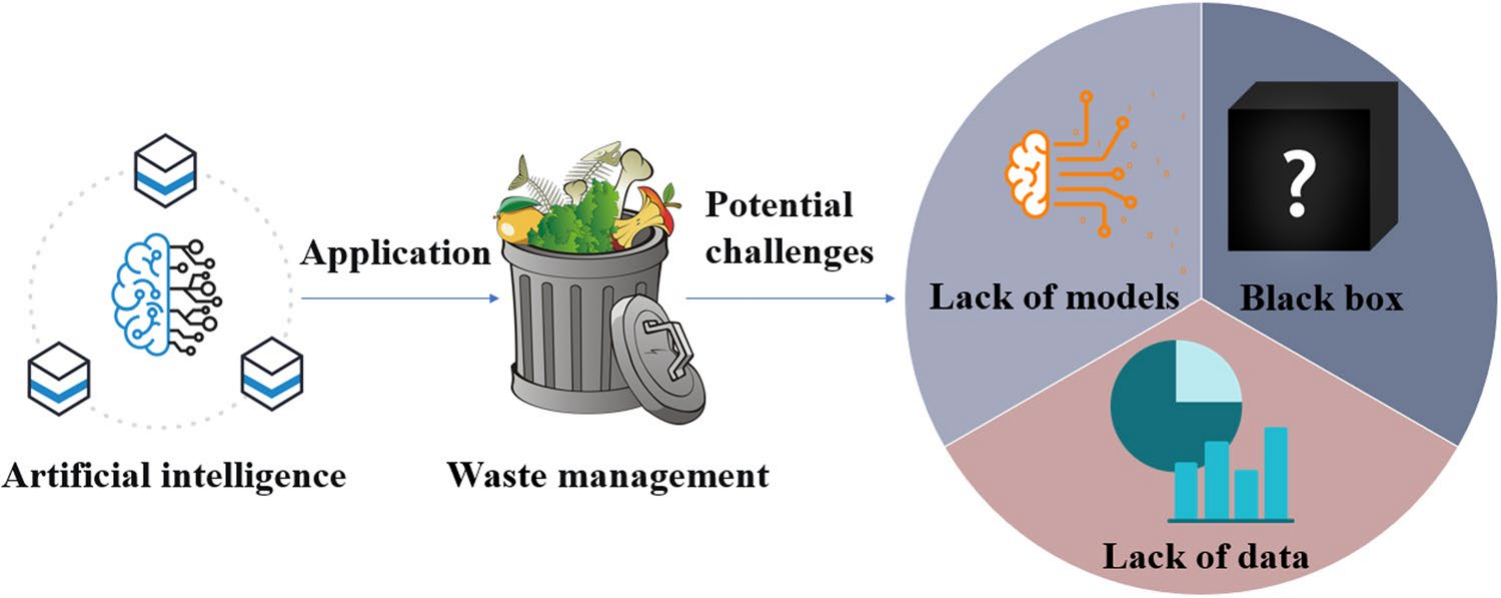
Three challenges of artificial intelligence in waste management. Implementing artificial intelligence in waste management can be summarized as black box problems, a lack of data, and a shortage of suitable models. Black boxes refer to the complexity of artificial intelligence models, which makes it difficult for researchers to understand their mechanisms. Lack of data refers to the scarcity and unreliability of data in the waste management industry, making it challenging to train artificial intelligence models. Finally, the lack of suitable models means that most existing applications of artificial intelligence in waste management rely on preexisting models rather than custom models explicitly developed for waste management

It can be concluded that implementing artificial intelligence in waste management has the potential to enhance efficiency and decrease costs. However, challenges like black box problems, inadequate data, and a lack of customized artificial intelligence models for waste management still exist. Figure 6 illustrates three potential obstacles that may arise in the application of artificial intelligence in waste management.

## Limitation and prospects 

In terms of artificial intelligence to optimize waste logistics and transportation, Zhang et al. (2020) pointed out that when the back-off algorithm optimizes the path of garbage collection vehicles, there is a limitation that the collection vehicles are at a fixed speed, which is impossible in real life.

In terms of artificial intelligence to identify illegal dumping, Takahashi et al. (2022) proposed that the faster regions with convolutional neural network features model can only be used to identify waste near rivers and cannot be used to identify waste in other complex areas such as cities. Moreover, there are also certain limitations in using the single-shot detector algorithm to identify waste in drone images. The algorithm cannot identify waste in covered areas such as woods (Youme et al. 2021). Additionally, Padubidri et al. (2022) proposed a method using a deep learning model to identify illegal dumping sites in high-resolution aerial images, which may suffer from identification errors and is not suitable for identifying low-resolution aerial images.

Regarding the use of artificial intelligence for waste identification and sorting, the waste images used to train transfer learning and convolutional neural network models for waste classification are much less complex than real-world waste, leading to reduced accuracy rates in practical applications (Zhang et al. 2021a). Additionally, researchers have explored using image data augmentation and transfer learning to identify and classify construction waste. Increasing the amount of data reduces the quality of the model. Considering the picture quality, it is best to avoid sunrise and sunset when collecting pictures. In addition, waste detection and classification systems based on convolutional neural networks have high hardware costs and energy consumption limitations (Ziouzios et al. 2022).

Learn more about the mechanics of artificial intelligence models. Because of the black box characteristics of artificial intelligence models, it is difficult for people to understand the mechanism of artificial intelligence models (Guo et al. 2021). Now, there are some methods to explain the influence of input variables on artificial intelligence models, such as using visualization technology; Selvaraju et al. (2020) use class activation mapping to explain the mechanism of convolutional neural network. However, only a few methods have been applied to explain the mechanisms of artificial intelligence models. In the future, researchers could develop more ways to interpret artificial intelligence models for greater understanding (Lin et al. 2022).

Combine artificial intelligence models with other technologies (Guo et al. 2021). For example, combining artificial intelligence models and Internet of things (IoT) technology allows artificial intelligence to be better applied to waste management (Guo et al. 2021). In addition, it is also possible to combine artificial intelligence models and data science. This can provide more high-quality data for training artificial intelligence models to improve the quality of the model (Lin et al. 2022).

The combined use of multiple artificial intelligence models is an inevitable trend. Artificial intelligence models include but are not limited to convolutional neural networks, residual network models, and gradient enhancement regression models. Most existing research focuses on a single artificial intelligence model for waste management. Using a combination of multiple models for waste management has better accuracy than a single model and effectively prevents overfitting (Guo et al. 2021). This can better solve the service management problem.

In summary, the limitations of artificial intelligence in waste management are primarily related to its difficulty in practical operation compared to theory, the limited scope of application, and lower efficiency in practical use. The potential for future progress lies in comprehensively understanding the mechanisms behind artificial intelligence models, combining artificial intelligence with other technologies, and using multiple models, such as convolutional neural network, residual network model, and gradient enhancement regression model .

## Conclusion

Waste disposal is inefficient, leading to severe environmental pollution, high costs, and a lack of leadership in the disposal process. Waste management is a challenge for both developed and developing countries. However, artificial intelligence can improve treatment efficiency, reduce environmental damage, and provide computational solutions for smarter waste management. This review is divided into nine sections, including definitions and the application of artificial intelligence in waste management. It highlights the potential impact of artificial intelligence on waste management, with practical applications such as smart bin systems, waste-sorting robots, and predictive waste tracking models. Artificial intelligence can also assist in managing hazardous waste, reducing illegal dumping, and recovering valuable resources from the waste stream. Additionally, artificial intelligence can aid public health interventions, including medical waste disposal and pandemic response.

The paper examines the impact of artificial intelligence on waste logistics and transportation, including reducing distance, cost, and collection time and improving collection efficiency. Although some algorithms have limitations, artificial intelligence can optimize waste treatment methods such as recycling, composting, landfill, and incineration. Machine learning, artificial intelligence, and deep learning techniques can improve waste classification, predict heavy metal levels in compost, and model waste incineration processes. Environmental variables such as temperature, humidity, and light can affect waste management artificial intelligence systems, causing fluctuations. Despite these challenges, artificial intelligence can change how people deal with waste, leading to a more sustainable future with efficient, economic, ecological, and intelligent waste management systems.
